# Both Movement-End and Task-End Are Critical for Error Feedback in Visuomotor Adaptation: A Behavioral Experiment

**DOI:** 10.1371/journal.pone.0055801

**Published:** 2013-02-05

**Authors:** Takumi Ishikawa, Yutaka Sakaguchi

**Affiliations:** Department of Information Media Systems, University of Electro-Communications, Tokyo, Japan; University of Milan, Italy

## Abstract

An important issue in motor learning/adaptation research is how the brain accepts the error information necessary for maintaining and improving task performance in a changing environment. The present study focuses on the effect of timing of error feedback. Previous research has demonstrated that adaptation to displacement of the visual field by prisms in a manual reaching task is significantly slowed by delayed visual feedback of the endpoint, suggesting that error feedback is most effective when given at the end of a movement. To further elucidate the brain mechanism by which error information is accepted in visuomotor adaptation, we tested whether error acceptance is linked to the end of a given task or to the end of an executed movement. We conducted a behavioral experiment using a virtual shooting task in which subjects controlled their wrist movements to meet a target with a cursor as accurately as possible. We manipulated the timing of visual feedback of the impact position so that it occurred either ahead of or behind the true time of impact. In another condition, the impact timing was explicitly indicated by an additional cue. The magnitude of the aftereffect significantly varied depending on the timing of feedback (p < 0.05, Friedman's Test). Interestingly, two distinct peaks of aftereffect were observed around movement-end and around task-end, irrespective of the existence of the timing cue. However, the peak around task-end was sharper when the timing cue was given. Our results demonstrate that the brain efficiently accepts error information at both movement-end and task-end, suggesting that two different learning mechanisms may underlie visuomotor transformation.

## Introduction

The brain updates motor memory so as to achieve a given task in a changing environment. In throwing a ball toward a visual target, for example, we can modify the throwing action as the weight of the ball is changed [1]. This motor adaptation is driven by the error signal or task outcome, information which is fed back during or after the task execution [2]–[3]. Considering that we can modify our movements according to the change in the relationship between visual information (i.e., target position) and motor action [4]–[25], the brain presumably updates the visuomotor transformation adaptively. The learning mechanism underlying this adaptation has been examined in a number of studies in which the visual environment is distorted by a wedge prism or virtual reality devices, typically while subjects perform a reaching [4]–[16] or shooting [17]–[24] task. When the endpoint (reaching) or impact point (shooting) is displaced, the endpoint error gradually decreases and the subject correctly reaches/shoots the target after a few dozen trials.

An important question is how the brain acquires the information required for regaining task performance. For prism adaptation in a manual reaching task, knowing the visual position of the endpoint is essential, and the endpoint error calculated from this information drives the adaptation. One important aspect of error feedback is its timing [11], [15], [16]. Kitazawa et al. [11] demonstrated that delayed visual feedback slowed prism adaptation for reaching movements. When visual presentation of the endpoint was delayed for more than 50 ms, the amount of aftereffect and the speed of adaptation diminished significantly. This suggests that the brain accepts error signals most effectively when they are synchronized with the end of reaching movements.

Recent studies replicated this effect. Tanaka et al. [15] showed that the efficiency of prism adaptation was degraded by adding artificial delay of 100 ms. They also reported that this degrade was consistently observed even when the subjects had been adapted to the delay of visual feedback and the amount of their subjective delay was significantly decreased (to about 40 ms). Thus, they concluded that the efficiency of prism adaptation was determined not by the subjective delay but by the physical delay. Honda et al. [16] showed that the delayed visual feedback degraded the visuomotor adaptation even when the visual shift was gradually increased so that the subject had difficulty being aware of the existence of the visual shift. In addition, they compared the magnitude of adaptation among no-delay, sudden-delay and adapted-delay conditions, where the visual shift was suddenly imposed in the adaptation trials under the sudden-delay condition while the visual shift was introduced from the beginning of the experimental session under the adapted-delay condition. In contrast to the study by Tanaka et al. [15], the degradation of adaptation in the sudden-delay condition was significantly alleviated under the adapted-delay condition. Honda et al. [16] pointed out that the temporal association between the motor action and its sensory consequence is an essential factor in visuomotor learning.

**Figure 1:**
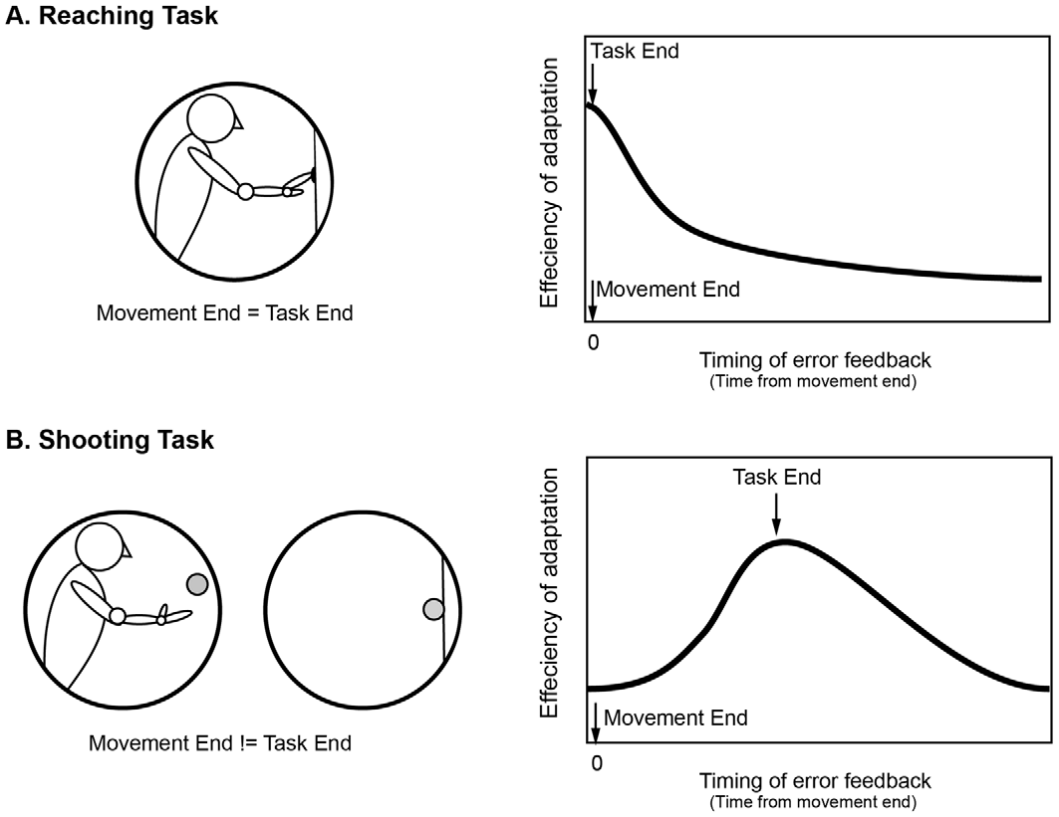
Working Hypothesis. A1: In a reaching task, the timing of task-end is synchronized with the end of body movement. The efficiency of visuomotor adaptation peaks immediately after movement-end (which is equivalent to the task-end), and diminishes uniformly with visual feedback delay. B1: In a shooting task, a ball hits a target some time after the shooter’s arm movement is complete; therefore, the timing of task-end is dissociated from the timing of movement-end. Our working hypothesis was that visuomotor adaptation should not exhibit peak efficiency immediately after movement-end, but at task-end (the time of impact). In other words, we predicted that efficiency would not diminish uniformly with delayed feedback.

In a shooting task in which a ball is thrown [17]–[24], a ball hits a target some time after it leaves the shooter’s hand, so that the timing of the task-end (i.e., time of impact) is dissociated from the timing of motor execution or the end of the body movement (i.e., time of action). An interesting question is whether error feedback is linked to "task-end" or "movement-end." Considering that the goal of adaptation is to maintain task performance, the timing of task-end, but not movement-end, may be critical for accepting feedback information, as we hypothesize in Figure 1. This can also be regarded as the association between motor action and its consequence [16].

To examine these possibilities, we conducted a behavioral experiment using a virtual shooting task in which subjects controlled their wrist movements to shoot a target on a screen by moving a cursor as accurately as possible. A visual shift was introduced by displacing the ball trajectory on the screen. The timing of visual feedback of the impact location was manipulated. The time from the throwing action (movement-end) to impact (or cursor speed) was also varied (600 ms or 1100 ms). We also ran an experiment in which the timing of impact was explicitly indicated by an additional timing cue to examine the effect of certainty of the impact timing. The magnitude of adaptation was based on the amount of aftereffect estimated from the adaptation curve. The results indicated that the amount of aftereffect significantly varied depending on the feedback timing. Specifically, the curve showed two distinct peaks around the end of wrist movement and around the time of impact. This result was consistently observed irrespective of the time from the end of the movement to impact (or the speed of the cursor). Additionally, the peak around task-end became sharper when the additional timing cue was introduced. These results suggest that the brain accepts error information at two distinct times, and that the task-end timing may be determined by prediction by some internal forward model (or temporal association between motor action and its sensory consequence).

A preliminary account of this study has been partly reported elsewhere [26], [27].

## Results

### Virtual Shooting Task and Control of Error Feedback Timing

The subjects’ task was to hit a target on the screen with a cursor whose motion was determined by their wrist movement. The subjects were asked to initiate wrist movement immediately after the target presentation within a required reaction time range. The cursor movement speed was fixed to one of two different values (independent of the wrist rotation speed): the time from the throwing action to impact (cursor Movement Time, cMT) was either 600 ms or 1100 ms. In the main experiment, cursor trajectory was not presented and only the impact point was displayed on the screen. Here, we manipulated the timing of error feedback. Under some conditions, visual information of the cursor endpoint was given later than task-end (i.e., expected impact timing), while under the other conditions, it was given in advance of task-end (see Materials and Methods for details).

### Reaction Time

Subjects made wrist movements within the required reaction time (i.e., 200 ms < RT< 400 ms) in more than 92% of the trials.

### Typical Experimental Results

**Figure 2:**
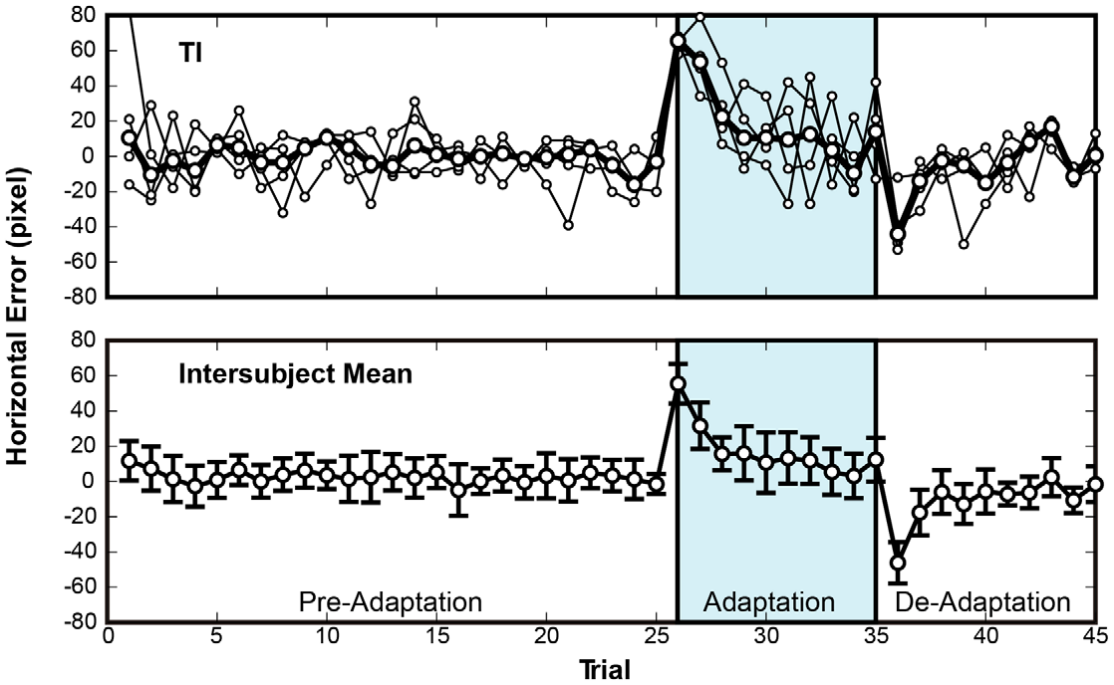
Adaptation curve from Experiment 1, 0-ms condition. The horizontal error is plotted against the sequence of trials. The upper and lower panels show the data for one typical subject (TI) and the average for all subjects, respectively. In the upper panel, the thin lines represent the results for four individual sessions and the bold line represents their median. Although inter-session variability was rather large, the median shows a typical adaptation curve. In the lower panel, the bold line shows the averages for all subjects, and the error bars show the standard deviation. The averaged learning curve is a classic adaptation curve.

Figure 2 shows the learning curve obtained for one subject (TI), and the averaged learning curve obtained for all subjects. In this figure, horizontal errors relative to the target are plotted against the sequence of trials. In the upper panel, each thin line shows the median horizontal errors under one delay condition, and the thick line represents the median of the thin lines. In the lower panel, the thick line shows the averaged learning curve and the error bars show the standard deviations for individual trials.

In the pre-adaptation period, the shooting errors were distributed around zero, indicating that the subjects successfully performed the virtual shooting task. In the adaptation period, errors occurred in the same direction as the visual displacement in the first trial, and decreased in subsequent trials. In the de-adaptation period, errors occurred in the opposite direction and again uniformly decreased over successive trials. Therefore, the visual distortion in our virtual shooting task resulted in a learning curve similar to those reported for prism adaptation experiments.

### Effect of Error Feedback Timing

**Figure 3:**
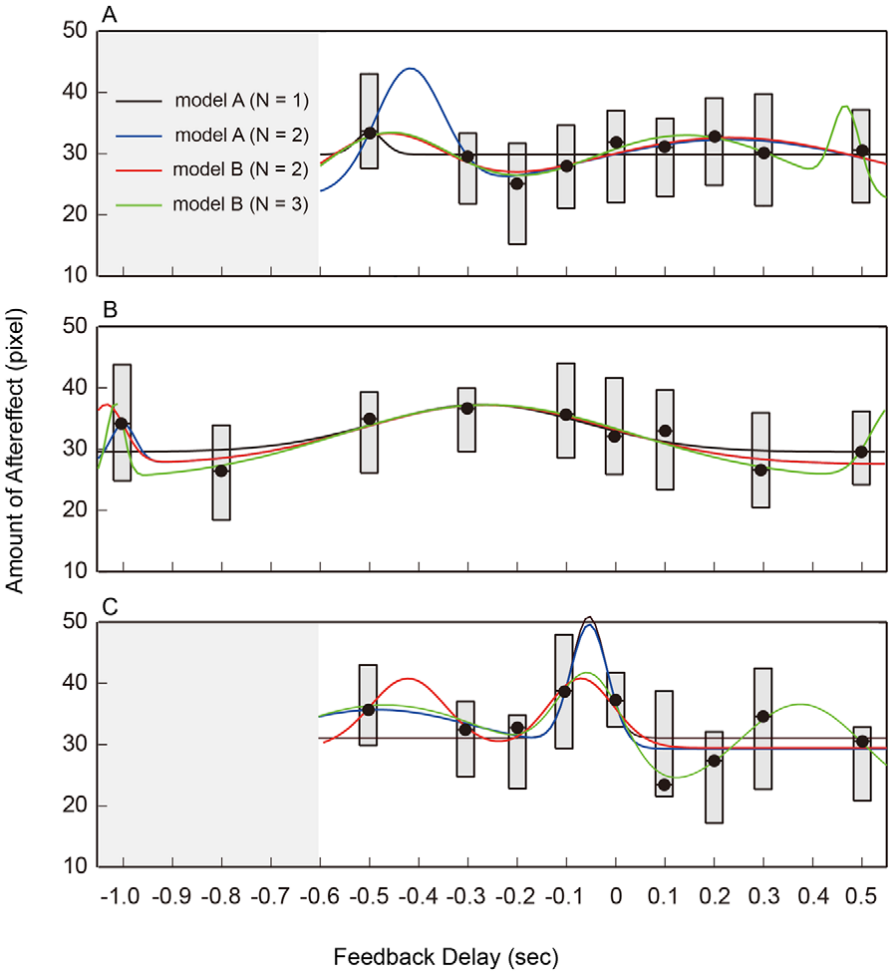
Experimental Results. The estimated aftereffect (*A_d_*: see Materials and Methods) is plotted against the delay conditions. The box plot shows the quartiles: thick segments indicate the medians, and the top and bottom of the boxes show the 25th and 75th percentiles. Line plots are the curves fitted using four different radial basis functions. A: Cursor movement time (cMT) was 600 ms and no timing cue was presented (Expt. 1), B: cMT was 1100 ms and no timing cue was presented (Expt. 2). C: cMT was 600 ms and the timing cue was presented (Expt. 3). In each case, radial basis curves with two basis functions seem to best fit the experimental result, implying that the relationship between the feedback delay and aftereffect had two peaks.

Figure 3 shows the time constant and the aftereffect of adaptation. The three panels show the quartile of the aftereffect (*A_d_*, see Materials and Methods) for all subjects in three experiments. In Experiment 1 (cMT 600 ms, no timing cue), the amount of aftereffect varied depending on the timing of feedback (Figure 3A). The aftereffect was large under the –500 ms condition (i.e., when feedback was given just after movement-end) and decreased under the –300 ms condition. It then increased again and peaked broadly across the 0–500 ms feedback delay range (i.e., around task-end). When the feedback was delayed 1000 ms, the aftereffect decreased again. The effect of feedback delay on the amount of aftereffect was statistically significant (*p* < 0.05, Friedman test (nonparametric one-factor repeated measures analysis of variance [ANOVA])). These results demonstrate that the efficiency of visuomotor adaptation varied depending on the timing of error feedback. We performed a post-hoc multiple comparison test on this result, but no significant difference was detected.

To further examine this result, we attempted to fit the relation between the feedback delay and amount of aftereffect with radial basis functions. The colored lines in Figure 3 show four approximation curves that have one, two, two and three basis functions, respectively (see Materials and Methods for detail). The relationship was well approximated by the curves with two basis functions, but not by the curve with a single basis function. The curve with three basis functions also fits well the relationship, but is apparently no better than the curves with two-basis functions. This indicates that the delay–aftereffect relationship has two distinct peaks.

The centers of the basis functions were located at –420 and 230 ms (model A) and –470 and 230 ms (model B), which implies that adaptation efficiency increased most at about 30–80 ms after movement-end and at about 230 ms after task-end. Moreover, the widths (2σ) of the basis functions were 140 and 600 ms (model A) and 240 and 600 ms (model B), meaning that the temporal window of error feedback for efficient adaptation is broader around the task-end than after the movement-end.

In Experiment 2 (cMT 1100 ms, no timing cue), we found similar results (Figure 3B). The amount of aftereffect was slightly larger just after movement-end (–1000 ms condition), but diminished under the –800 ms condition. It increased again and peaked around the –100 ms feedback delay condition. The effect of feedback delay on the amount of aftereffect was statistically significant (*p* < 0.05, Friedman test). The fitting curves are indicated by the colored lines (Figure 3B). Again, the curves with two or three basis functions well approximated the relationship, but those with two basis functions sufficiently captured the global nature of the relationship. The centers of the basis functions in the two-basis case were –1000 and –260 ms (model A) and –1030 and –260 ms (model B), showing that the adaptation efficiency improved around the movement-end and some time before the task-end. On the other hand, the widths of the basis functions were 50 and 500 ms (model A) and 70 and 500 ms (model B), which shows again that the time window of the efficient adaptation was broader around the task-end than after the movement-end.

Results for Experiment 3 (cMT 600 ms, with timing cue) also revealed a similar pattern (Figure 3C). The aftereffect was larger just after movement-end (the –500 ms condition), decreased under the –300 and –200 ms conditions, and then increased again and peaked broadly around 0 ms feedback delay. The results for the 0–500 ms conditions, however, were somewhat more variable than those in Experiment 1; in particular, the peak around the 0 ms condition seemed sharper in Experiment 3. The effect of the feedback delay was significant (*p* < 0.05, Friedman test). Again, curves with two basis functions well and sufficiently fit the data. The centers of the basis functions in the two-basis case were –480 and –50 ms (model A) and –420 and –70 ms (model B), and their widths were 380 and 70 ms (model A) and 150 and 130 ms (model B), showing a tendency consistent with that for the previous results. However, we note that the second peak (around the task-end) was remarkably narrower in this experiment (70–130 ms vs. 600 ms in Expt. 1 and 500 ms in Expt. 2), implying that that the temporal window of efficient adaptation was narrower when the timing cue was added.

Although the results of above experiments show that the amount of aftereffect significantly depended on the feedback delay and increased around movement-end and task-end, the result seems somewhat statistically weak for the decisive evidence. This weakness stems from the large variance of the amount of aftereffect, which was fundamentally due to the variability of the movement (i.e., throwing movement is less stable than reaching). In order to have additional evidence to support our view, we ran a supplementary experiment with a modified task. It was still a shooting task but was like a remote pointing task where subjects were asked to point the target with their right arm. The cursor started off at the end of the pointing action and moved to the pointed location in 600 ms (see Materials and Methods for detail). This task could be performed more easily than the original shooting task, which led to improving the movement stability. Actually, the fitting error of adaptation curve (RMS) was reduced to one half of that of the original task. We compared the amount of aftereffect among four delay conditions in this experiment, because here we are mainly interested in whether or not two distinct peaks were found just after the movement end and the task end.

**Figure 4:**
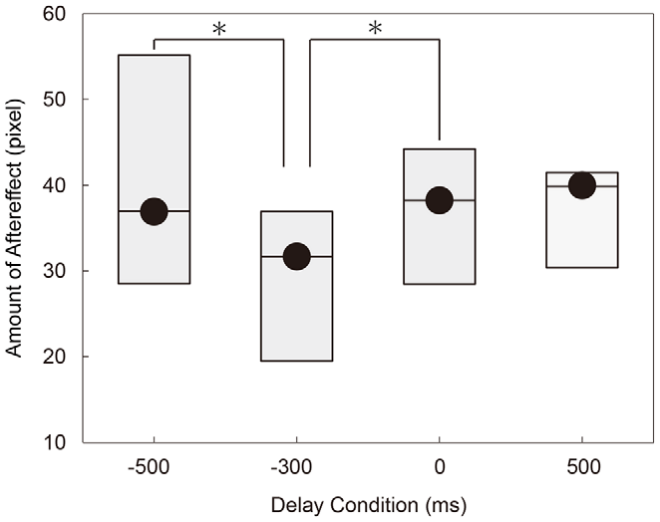
Results of Supplementary Experiment. The estimated aftereffect (*A_d_*: see Materials and Methods) is plotted for four delay conditions. The box plot shows the quartiles: thick segments indicate the medians, and the top and bottom of the boxes show the 25th and 75th percentiles. Asterisks indicate significant difference (*p* < 0.05) detected by a post-hoc multiple comparison test (Scheffe’s method). The amount of after effect was significantly larger when the feedback was given 100 ms after the movement offset and at the task end, compared to the case that it was given 300 ms before the task end.

Figure 4 summarizes the result. As in Figure 3, the quartiles of the aftereffect were shown for four delay conditions. Apparently, the aftereffect had larger values at just after the movement end and at the task end, compared to the other two conditions. The effect of feedback delay on the amount of aftereffect was statistically significant (*p* < 0.05, Friedman test).A post-hoc multiple comparison test (Scheffé's method) showed that there was significant difference between -500 ms (movement end) and -300 ms conditions and between -300 ms and 0 ms (task end) conditions, supporting that the efficiency of visuomotor adaptation was increased at both movement end and task end.

## Discussion

In the present study, we examined the effect of error feedback timing on the efficiency of visuomotor adaptation in a virtual shooting task, and found that the amount of aftereffect depended significantly on the feedback delay and increased around movement-end and task-end. This tendency was consistently observed irrespective of the time between movement-end and task-end, and regardless of whether a timing cue signaled task-end. Our results suggest that the timing of error feedback significantly affected the efficiency of visuomotor adaptation, and that efficiency was enhanced both around movement-end and task-end. Moreover, these two peaks of the aftereffect differed in the magnitude; the first peak (just after movement-end) was steeper than the second peak (around task-end) and the second peak became a little sharper when the timing cue was introduced.

Kitazawa et al. [11] showed that adaptation efficiency or the amount of prism adaptation diminished significantly when feedback was delayed more than 50 ms. In their study, moreover, efficiency reduced almost uniformly as feedback delay increased. In the present study, in contrast, we did not observe a uniform change in efficiency as feedback timing varied. Rather, we found two distinct peaks in efficiency, after movement-end and around task-end. This tendency was not confirmed by post hoc multi-comparison tests (but confirmed in the supplementary experiment), but occurred consistently under three different conditions. Therefore, we believe these results suggest that both movement-end and task-end are critical times for visuomotor adaptation.

Here, we discuss the mechanism underlying the acceptance of error information in visuomotor adaptation. There are at least three possible ways in which the brain determines the timing of error acceptance. Error acceptance timing may be: 1) locked to the time of motor command generation; 2) specified by the sensory information accompanying movement-end or task-end; or 3) predicted within the brain. In shooting, the brain has to map the visual information of the target position into the motor command for appropriate throwing action, and this mapping has to be updated in the process of prism adaptation. This mapping is called an “inverse model” in computational neuroscience because it transfers the desired result (i.e., target position to hit) to the cause (i.e., motor command) [2], [3], [28]–[30], but its neural substrates have not yet been revealed. For example, the endpoint error when using visual information is simply the difference between the expected endpoint and actual endpoint, and not the direct teacher signal for updating the motor command; it is unclear how the visual error information is used to update the motor commands. Here, we do not go into the details of such a learning mechanism of the inverse model. However, if the brain updates the inverse model according to the outcome of the motor commands, the brain has to memorize the motor commands at least until the error information is fed back. In addition, it has been discussed that the brain has a “forward model”, which predicts the result (i.e., impact position and its timing) from the cause (i.e., motor command) [2], [3], [31]. The visual information given at the task-end can be the teacher signal for updating the forward model. Keeping these ideas (i.e., motor memory and prediction) in mind, we discuss the above three views.

The first possibility implies that the error signal should be imposed immediately after the command generation. This is related to the view that the cerebellum has a mechanism compensating the temporal interval between the motor command and its sensory consequence [32]. Considering that cerebellar patients did not show prism adaptation [17]–[19] and that the cerebellum plays an important role in motor execution and timing control [33]–[35], the cerebellum is an important organization for prism adaptation. In addition, it has been reported that long-term depression found in the cerebellum is most efficient when climbing fiber signals (i.e., sensory error signals) are delayed by about 250 ms with respect to the parallel-fiber signals (i.e., motor commands) [36]–[38]. Therefore, if this built-in mechanism is used for adaptation, the adaptation occurs without any motor memory or temporal prediction. This view can simply explain the result obtained by Kitazawa et al. [11] that delayed visual feedback slows the prism adaptation in a reaching task.

The second view, to the contrary, assumes that the brain can wait for the external cue providing the error information. This means that the brain has to memorize “cause” information until the “effect” appears. Taking this view, however, the brain does not need to know the timing of the effect; if the brain learns the association between the effect (target impact) and accompanying sensory event, the sensory events give the time to catch the error information. Thus, the second view requires motor memory but no temporal prediction.

The third possibility, in contrast, assumes that the brain predicts the timing of the effect. In other words, the brain opens the gate for error acceptance according to an internal timer. Therefore, the third view assumes both motor memory and temporal prediction. Note that in this study, the subjects practiced the task several hundred times in the situation that the cursor movement was completely visible. Thus, it is plausible that they had gained the forward model of the cursor movement and could anticipate the task-end timing (i.e., impact timing) even when the cursor was not visible. Of course, this can be regarded as the subjects learning the association between the motor command and sensory consequence [16]. Below, we examine the above three possibilities according to this difference.

First, it is plausible that the first peak is linked to motor execution or motor command generation (i.e., the first hypothesis). Because the timing of motor execution or movement-end can be detected directly and precisely, the brain could strictly determine the timing of error acceptance (because the brain need not predict the timing of error information) if this cue is used. This would explain why the first peak was sharp. On the other hand, the second peak might be caused by the prediction of task-end (i.e., the third hypothesis). If no specific sensory cue is given, the timing must be predicted or expected, and consequently, it would be expected to be less precise. If the brain determines the timing of error acceptance based on prediction, the time range for error acceptance would be expected to broaden, to compensate for uncertainty or imprecision. This may explain why the peak around task-end was broader. In addition, the second peak became steeper when the task-end timing was explicitly indicated (in Experiment 3), suggesting that a sensory cue indicating or helping to estimate the timing of task-end would narrow the time range for error acceptance. This is in accordance with the notion that sensory cues may also be involved in determining error acceptance timing, supporting the second hypothesis.

At present, therefore, we consider that no single mechanism determines the timing of error acceptance. In other words, we speculate that multiple error acceptance mechanisms must be involved in visuomotor adaptation. As mentioned above, these mechanisms use different functions (e.g., motor memory and temporal prediction) of the brain system. Therefore, our speculation might be tested employing brain imaging techniques.

## Concluding remarks

In the present study, the efficiency of visuomotor adaptation depended on the timing of error feedback and it increased around both movement-end and task-end, suggesting that two different learning mechanisms may underlie visuomotor transformation. We also suggest that expectation of the task-end timing might be a cue for accepting error information in sensory signals that aids visuomotor adaptation. This expectation could be based on the association (or forward model) between the motor command (i.e., cause) and its consequence (i.e., effect). Future studies are needed to determine which neural structures are responsible for these mechanisms.

## Materials and Methods

### Subjects

This research was approved by the authors' institutional review board. All subjects received an adequate explanation of the merits and demerits of participation in this research. All subjects were paid 1000 Japanese Yen (about 12 US dollars) for 1 hour, and we obtained an informed consent form from all subjects. All subjects had normal or corrected-to-normal visual acuity and no significant neurological history. All subjects (except author TI) were naive as to the purpose of the experiments.

Twenty subjects (aged 18–30 years, two females and 18 males) participated in Experiment 1, all of whom except one male subject were right-handed. Twenty subjects (aged 18–28 years, two females and 18 males) participated in Experiment 2; five of these subjects had participated in Experiment 1, and all except two males were right-handed. Eleven subjects (aged 18–31 years, 11 males) participated in Experiment 3, all of whom except three males were right-handed. Four of these subjects had participated in another experiment.

### Apparatus

**Figure 5:**
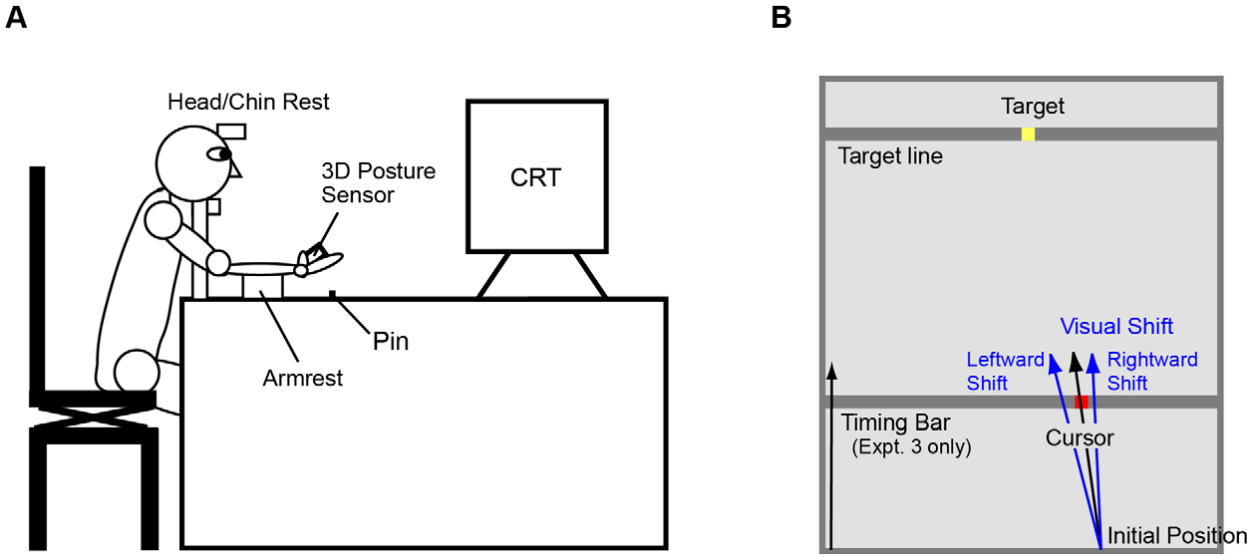
Experimental Setup. A: Subjects were seated facing a tangent 21-inch color CRT monitor with their heads restrained by a chin rest. Subjects put their right arms on an armrest with the right palm upturned and gripped a 3D posture sensor. Subjects were not able to observe their own movements. The 3D posture sensor measured Euler angles of subjects’ wrist movements. The pitch angle was referred to as the flexion angle and used to trigger the cursor movement, while the roll angle was referred to as the pronation/supination angle and determined the direction of cursor movement.B: The diagram shows the display on the CRT monitor. The area of the task field was 675×550 pixels. The target appeared on the target line, located 650 pixels from the bottom of the monitor. The initial position of the cursor was 100 pixels to the right of the bottom center of the monitor, and cursor movement was initiated by flexion movement of the wrist. The direction of cursor movement was controlled by the pronation/supination angle, but the vertical velocity was fixed throughout the experimental session. Visual displacement was introduced by rotating the direction of cursor movement and, as a result, the position of the cursor endpoint was displaced in a horizontal direction. Note that in Experiments 1 and 2, the cursor was displayed only at the target line (i.e., impact position), although it was displayed from the initial position to the impact position in the practice session. In Experiment 3, the cursor was not displayed; instead, a timing bar was presented that indicated the vertical position of the cursor.

Each subject was seated facing a tangent 21-inch color cathode ray tube (CRT) monitor (GDM-F500, refresh rate: 100 Hz; Sony Corp., Tokyo) placed 800 mm from the eyes, with his/her head restrained by a chin rest (Fig. 5A). The screen size was 1024×768 pixels. Subjects put their right arms on an armrest with the palm upturned, and gripped a three-dimensional (3D) posture sensor (MTx-28A53G25, Xsens Technologies B.V., Enschede). The armrest was positioned 250 mm ahead and 250 mm to the right of the midsagittal plane (Fig. 5A). Subjects were asked to touch a pin using the middle finger of their right hand. This was the initial posture of each trial. The pin was positioned 350 mm ahead and 350 mm to the right of the midsagittal plane. Subjects were not able to observe their own movements. The 3D posture sensor measured Euler angles (i.e., roll, pitch, and yaw) of subjects’ wrist movements with time resolution of 5 ms. An IBM AT compatible personal computer acquired posture data through a universal serial bus (USB) interface. In this experiment, the pitch angle of wrist movements was referred to as the flexion angle. Similarly, the roll angle was referred to as the pronation/supination angle.

Figure 5B shows the view of the task field on the monitor. The area was 27 cm×22 cm (675×550 pixels). The target appeared on the target line, located 26 cm (650 pixels) from the bottom of the monitor. The direction of cursor movement was controlled by subjects' wrist movements (pronation or supination). The initial position of the cursor was 4 cm (100 pixels) to the right of the bottom center of the monitor. Cursor movement was initiated by flexion movement of the wrist. Specifically, cursor movement started within 20 ms of the angular velocity of the flexion movement reaching its peak value. Note that the vertical velocity of the cursor was constant (600 ms or 1100 ms, from the initial position to the target line) regardless of the joint velocity or the direction of wrist movement. In Experiment 3, subjects were informed that the vertical position of the cursor was indicated by a horizontal bar sliding in the vertical direction. (The cursor itself was invisible.) The size of the tracking bar was 0.4 cm×22 cm.

### General task procedure

**Figure 6:**
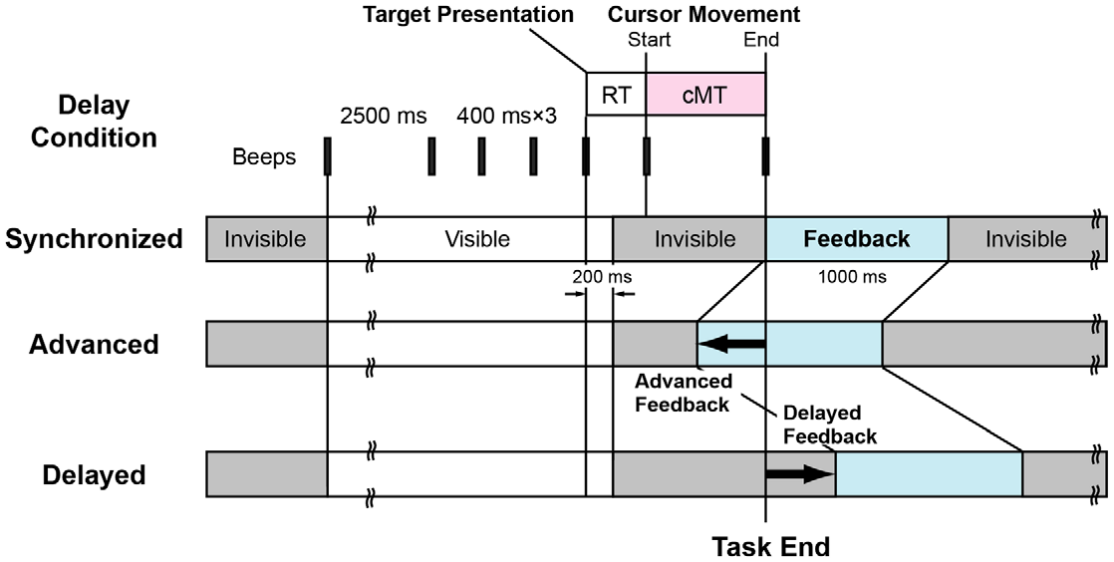
Task Schedule. Each trial started with a beep sound and the appearance of a target line. After a 2500-ms pause, three intermediate-tone beeps were given at 400-ms intervals, followed by a high-tone beep, and the appearance of a target at a random position on the target line. The subject began wrist movement immediately after target presentation so as to hit the target with a cursor. The computer program monitored wrist movements, detected their peak angular velocity and initiated the cursor movement. Timing of visual feedback differed among the delay conditions. Under some conditions, visual information was given later than task-end, while under the other conditions, it was given in advance of task-end.

Figure 6 illustrates the general experimental procedure. Each trial started with a beep sound (10 ms, 450 Hz), and the appearance of a target line. After a pause of 2500 ms, three intermediate-tone beeps (10 ms, 600 Hz) were given at 400-ms intervals, followed by a high-tone beep (10 ms, 1000 Hz). At the onset of the high-tone beep, the target appeared at a random position on the target line, within a 40-pixel range around the center. The subject was asked to maintain the initial posture until the target was displayed, and to initiate wrist movement immediately after the target presentation so as to hit the target with the cursor. An alert message was displayed at the end of the trial if reaction time was less than 200 ms or greater than 400 ms. The computer program monitored wrist movements in a real-time manner, detected their peak angular velocity and initiated cursor movement as explained above. Note that the average wrist movement time was about 120 ms, and cursor movement initiation preceded each movement’s end by about 30 ms. Subjects were asked to maintain their final wrist position until that trial ended.

In experimental sessions, subjects were not able to observe the trajectory of the cursor (although they could see the trajectory in a practice session, as mentioned below). Note that the frame and target line were always visible. In Experiment 3, subjects were able to observe the vertical position of the cursor by the timing bar at any time.

The vertical velocity of the cursor movement was fixed throughout the experimental sessions. When the cursor reached the target line (i.e., at task-end), a high-tone beep (10 ms, 1000 Hz) was sounded, and the target and cursor were displayed. Visual displacement was introduced by rotating the direction of cursor movement, and as a result, the position of the cursor endpoint was displaced in a horizontal direction. The amount of displacement was 50 pixels on the target line. The timing of visual feedback differed among the delay conditions. Under some conditions, visual information was given later than task-end, while under the other conditions, it was given in advance of task-end (see below for details of the conditions). Each trial was terminated 7 s (Experiments 1 and 3) or 7.5 s (Experiment 2) after it started.

Subjects participated in the study for 7 days. On the 1st day, subjects practiced the virtual shooting task. Subjects completed more than 300 practice trials (50 trials/block×6 blocks), which took about 1 hour. In the practice trials, the entire trajectory of all cursor movements was presented to the subjects.

The main experiment began on the 2nd day. An experimental session consisted of 45 trials. Each session was composed of three blocks. In the first block (pre-adaptation block: 25 trials), neither a visual shift nor feedback delay was given. In the second block (adaptation block: 10 trials), a visual shift and a feedback delay (specified by each experimental condition) were introduced. In the third block (de-adaptation block: 10 trials), both the visual shift and feedback delay were eliminated. It took about 7 minutes to complete one session, and a subject performed six sessions per day, so that the experimental session finished within 1 hour.

Each subject performed the task four times (i.e., two times for rightward shift and two times for leftward shift) for each delay condition, i.e., a total of 36 ( =  4×9) experimental sessions. It thus took 6 days to complete all sessions. The order of presentation of the different experimental conditions was randomly determined.

### Feedback Timing Conditions

In Experiments 1 and 3, the cursor reached the target line 600 ms after movement onset. The amount of visual feedback delay/advance was chosen from nine values: –500, –300, –200, –100, 0, 100, 200, 300 and 500 ms. We referred to conditions with negative, zero and positive values as "advanced," "synchronized" and "delayed," respectively. In Experiment 2, the cursor reached the target 1100 ms after the movement onset. The amount of visual feedback delay/advance was chosen from nine values: –1000, –800, –500, –300, –100, 0, 100, 300 and 500 ms.

### Data analysis

The authors fitted the learning curve with two exponential functions: 
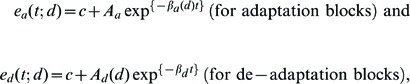
where *e_a_(t; d)* and *e_d_(t; d)* represent the horizontal error for trial *t* with feedback delay *d*. *A_a_* and *A_d_* represented the amplitude, and *b_a_* and *b_d_* the inverse time constant of the learning curves, and *c* is a constant representing the bias of individual subjects. Here, we note that we postulated that *b_a_* and *A_d_* depend on the delay conditions, while *A_a_* and *b_d_* would be common to all delay conditions.

To examine the effect of delay conditions on the efficiency of adaptation, we performed statistical tests for estimated aftereffect *A_d_*. We adopted a Friedman test (nonparametric one-factor repeated measures ANOVA) in the present study because we could not guarantee the equalities of variance.

To examine the existence of multiple peaks, we further attempted to approximate the feedback-delay vs. aftereffect relation using a radial basis function. The function used in the approximation was



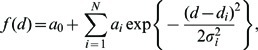
(model A)


where *N* is the number of basis functions. Parameters *a*_0_, *a_i_*, *d_i_*, and σ*_i_* were estimated by minimizing the sum of squared error between *f(d)* and the inter-subject median of aftereffect *A_d_*(*d*) using the “lsqcurvefit” function in the optimization toolbox of MATLAB. To obtain meaningful results, we set lower and upper bounds for each parameter. Note that the number of parameters is given by *N_p_*  =  1+3*N* while the number of data *N_d_* is nine (i.e., the number of delay conditions). Because *N_p_* should be less than the number of data *N_d_* for meaningful estimation, we could estimate the parameters only in the cases of *N*  =  1 and 2 (i.e., *N_p_*  =  4 and 7, respectively). Because we wanted to know the approximation performance also in the case of *N*  =  3, we also used the function



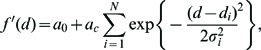
(model B)


where *a_c_* is a common weight for different basis functions. In this formulation, the number of parameters *N_p_* is given by 2+2*N* and we can estimate the parameters for *N*  =  1, 2 and 3 (note that this is the same as model A when *N*  =  1).

In total, we used four approximation functions, *N*  =  1 and 2 using model A and *N*  =  2 and 3 using model B.

We attempted calculating Akaike's information criterion for these models assuming a Gaussian error distribution, although it is in principle inappropriate to use this criterion here because we could not assume a specific probabilistic distribution (which is why we adopted non-parametric test) and thus could not calculate the likelihood function. The result partly supported that the fitting with two-basis functions was the best, but concrete results are not given in the text.

### Supplementary experiment

Supplementary experiment was run with the same experimental apparatus and procedure, except the points described below. The most significant difference was the subjects’ task. In this task, the subjects were asked to move the right forearm to point the target on the display, with their elbow supported on the desk. This movement could be achieved mainly by a flex movement of elbow joint, which helped the subjects to do the task more easily and intuitively. The cursor was initially located at the right side of the target line, and it started to move synchronized with the forearm movement, moved horizontally and stopped at the pointed position. The cursor movement time was fixed to 600 ms, and thus the cursor movement speed varied dependent on the pointed position. Therefore, the task itself was still a shooting task, but it was like a remote pointing task. In each trial, the subject was asked to maintain the initial posture until the target was displayed on the target line, and to initiate the movement immediately after the target presentation. The computer program initiated cursor movement when the arm movement was terminated (i.e., the angular shift between the successive sampling ticks became less than 1 degree). The average movement time was about 110 ms, but we should note that the movement time depended on the target position. Subjects were asked to maintain their final wrist position until that trial ended. In experimental sessions, subjects were not able to observe the trajectory of the cursor although they could see the trajectory in the practice session.

Visual displacement was introduced simply by shifting the endpoint of the cursor movement because the cursor movement was displayed only at the endpoint. The amount of displacement was 60 pixels. The timing of visual feedback was chosen from following four conditions. 100, 300, 600, and 1100 ms after the movement offset, that is, amount of visual feedback delay/advance was -500, -300, 0 and 500 ms respectively.

It took three session days (1 hour for each day) to finish. On the 1st day, subjects practiced the task by completing more than 250 practice trials, where the entire trajectory of all cursor movements was presented to the subjects. The main experiment began on the 2nd day. Each subject performed the task four times (i.e., two times for rightward shift and two times for leftward shift) for four delay conditions, i.e., a total of 16 ( =  4×4) experimental sessions. Eleven participants took part in this experiment.

## References

[1] Bingham GP, Schmidt RC, Rosenblum LD (1989) Hefting for a maximum distance throw: a smart perceptual mechanism. J Exp Psychol Hum Percept Perform 15: 507–528. 2527959 10.1037//0096-1523.15.3.507

[2] Shadmehr R, Smith MA, Krakauer JW (2010) Error correction, sensory prediction, and adaptation in motor control. Annu Rev Neurosci 33: 89–108. 20367317 10.1146/annurev-neuro-060909-153135

[3] Wolpert DM, Ghahramani Z (2000) Computational principles of movement neuroscience. Nat Neurosci 3 Suppl: 1212–1217 10.1038/81497 11127840

[4] Imamizu H, Shimojo S (1995) The locus of visual-motor learning at the task or manipulator level: implications from intermanual transfer. J Exp Psychol Hum Percept Perform 21: 719–733. 7643045 10.1037//0096-1523.21.4.719

[5] Stratton GM (1896) Some preliminary experiments on vision without inversion of the retinal image. Psychological Review 3: 611–617.

[6] Newport R, Jackson SR (2006) Posterior parietal cortex and the dissociable components of prism adaptation. Neuropsychologia 44: 2757–2765. 16504222 10.1016/j.neuropsychologia.2006.01.007

[7] Cressman EK, Henriques DY (2010) Reach adaptation and proprioceptive recalibration following exposure to misaligned sensory input. J Neurophysiol 103: 1888–1895. 20130036 10.1152/jn.01002.2009

[8] Kitazawa S, Yin PB (2002) Prism adaptation with delayed visual error signals in the monkey. Exp Brain Res 144: 258–261. 12012163 10.1007/s00221-002-1089-6

[9] Yin PB, Kitazawa S (2001) Long-lasting aftereffects of prism adaptation in the monkey. Exp Brain Res 141: 250–253. 11713636 10.1007/s002210100892

[10] Kitazawa S, Kimura T, Uka T (1997) Prism adaptation of reaching movements: specificity for the velocity of reaching. J Neurosci 17: 1481–1492. 9006989 10.1523/JNEUROSCI.17-04-01481.1997 PMC6793717

[11] Kitazawa S, Kohno T, Uka T (1995) Effects of delayed visual information on the rate and amount of prism adaptation in the human. J Neurosci 15: 7644–7652. 7472515 10.1523/JNEUROSCI.15-11-07644.1995 PMC6578075

[12] Baizer JS, Kralj-Hans I, Glickstein M (1999) Cerebellar lesions and prism adaptation in macaque monkeys. J Neurophysiol 81: 1960–1965. 10200230 10.1152/jn.1999.81.4.1960

[13] Tseng YW, Diedrichsen J, Krakauer JW, Shadmehr R, Bastian AJ (2007) Sensory prediction errors drive cerebellum-dependent adaptation of reaching. J Neurophysiol 98: 54–62. 17507504 10.1152/jn.00266.2007

[14] Wong AL, Shelhamer M (2011) Sensorimotor adaptation error signals are derived from realistic predictions of movement outcomes. J Neurophysiol 105: 1130–1140. 21123665 10.1152/jn.00394.2010 PMC3074418

[15] Tanaka H, Homma K, Imamizu H (2011) Physical delay but not subjective delay determines learning rate in prism adaptation. Exp Brain Res 208: 257–268. 21076819 10.1007/s00221-010-2476-z

[16] Honda T, Hirashima M, Nozaki D (2012) Adaptation to visual feedback delay influences visuomotor learning. PLoS One 7: e37900. 22666408 10.1371/journal.pone.0037900 PMC3364281

[17] Martin TA, Keating JG, Goodkin HP, Bastian AJ, Thach WT (1996) Throwing while looking through prisms. I. Focal olivocerebellar lesions impair adaptation. Brain 119 ( Pt 4): 1183–1198. 8813282 10.1093/brain/119.4.1183

[18] Martin TA, Keating JG, Goodkin HP, Bastian AJ, Thach WT (1996) Throwing while looking through prisms. II. Specificity and storage of multiple gaze-throw calibrations. Brain 119 ( Pt 4): 1199–1211. 8813283 10.1093/brain/119.4.1199

[19] Thach WT (1998) A role for the cerebellum in learning movement coordination. Neurobiol Learn Mem 70: 177–188. 9753595 10.1006/nlme.1998.3846

[20] Fernandez-Ruiz J, Velásquez-Perez L, Díaz R, Drucker-Colín R, Pérez-González R, et al (2007) Prism adaptation in spinocerebellar ataxia type 2. Neuropsychologia 45: 2692–2698. 17507059 10.1016/j.neuropsychologia.2007.04.006

[21] Fernandez-Ruiz J, Diaz R, Moreno-Briseño P, Campos-Romo A, Ojeda R (2006) Rapid topographical plasticity of the visuomotor spatial transformation. J Neurosci 26: 1986–1990. 16481431 10.1523/JNEUROSCI.4023-05.2006 PMC6674929

[22] Fernández-Ruiz J, Díaz R, Aguilar C, Hall-Haro C (2004) Decay of prism aftereffects under passive and active conditions. Brain Res Cogn Brain Res 20: 92–97. 15130593 10.1016/j.cogbrainres.2004.01.007

[23] Fernandez-Ruiz J, Diaz R, Hall-Haro C, Vergara P, Mischner J, et al (2003) Normal prism adaptation but reduced after-effect in basal ganglia disorders using a throwing task. Eur J Neurosci 18: 689–694. 12911765 10.1046/j.1460-9568.2003.02785.x

[24] Fernández-Ruiz J, Díaz R (1999) Prism adaptation and aftereffect: specifying the properties of a procedural memory system. Learn Mem 6: 47–53. 10355523 PMC311278

[25] Mazzoni P, Krakauer JW (2006) An implicit plan overrides an explicit strategy during visuomotor adaptation. J Neurosci 26: 3642–3645. 16597717 10.1523/JNEUROSCI.5317-05.2006 PMC6674132

[26] Ishikawa T, Sakaguchi Y (2011) Visual information of endpoint position is not required for prism adaptation of shooting task. ICONIP2011 95–102.

[27] Ishikawa T, Sakaguchi Y (2010) Effect of time difference between task-end and error-feedback on visuo-motor adaptation. Neuro2010 P2–j04.

[28] Wolpert DM, Ghahramani Z, Jordan MI (1995) An internal model for sensorimotor integration. Science 269: 1880–1882. 7569931 10.1126/science.7569931

[29] Kawato M (1990) Feedback-error-learning neural network for supervised motor learning. Advanced neural computers 6: 365–372.

[30] Doya K (1999) What are the computations of the cerebellum, the basal ganglia and the cerebral cortex? Neural Netw 12: 961–974. 12662639 10.1016/s0893-6080(99)00046-5

[31] Desmurget M, Grafton S (2000) Forward modeling allows feedback control for fast reaching movements. Trends Cogn Sci 4: 423–431. 11058820 10.1016/s1364-6613(00)01537-0

[32] Miall RC, Christensen LO, Cain O, Stanley J (2007) Disruption of state estimation in the human lateral cerebellum. PLoS Biol 5: e316. 18044990 10.1371/journal.pbio.0050316 PMC2229864

[33] Xu-Wilson M, Chen-Harris H, Zee DS, Shadmehr Ri (2009) Cerebellar contributions to adaptive control of saccades in humans. J Neurosci 29: 12930–12939. 19828807 10.1523/JNEUROSCI.3115-09.2009 PMC2994243

[34] Miall RC, Weir DJ, Wolpert DM, Stein JF (1993) Is the cerebellum a smith predictor. J Mot Behav 25: 203–216. 12581990 10.1080/00222895.1993.9942050

[35] Rao SM, Harrington DL, Haaland KY, Bobholz JA, Cox RW, et al (1997) Distributed neural systems underlying the timing of movements. J Neurosci 17: 5528–5535. 9204934 10.1523/JNEUROSCI.17-14-05528.1997 PMC6793838

[36] Chen C, Thompson R (1995) Temporal specificity of long-term depression in parallel fiber-purkinje synapses in rat cerebellar slice. Learn Mem 2: 185–198. 10467575 10.1101/lm.2.3-4.185

[37] Ito M, Sakurai M, Tongroach P (1982) Climbing fibre induced depression of both mossy fibre responsiveness and glutamate sensitivity of cerebellar Purkinje cells. J Physiol 324: 113–134. 7097592 10.1113/jphysiol.1982.sp014103 PMC1250696

[38] Ivry RB, Spencer RM, Zelaznik HN, Diedrichsen J (2002) The cerebellum and event timing. Ann N Y Acad Sci 978: 302–317. 12582062 10.1111/j.1749-6632.2002.tb07576.x

